# Adaptive multiscapes: an up-to-date metaphor to visualize molecular adaptation

**DOI:** 10.1186/s13062-017-0178-1

**Published:** 2017-02-28

**Authors:** Pablo Catalán, Clemente F. Arias, Jose A. Cuesta, Susanna Manrubia

**Affiliations:** 1 Grupo Interdisciplinar de Sistemas Complejos (GISC), Madrid, Spain; 2 Departamento de Matemáticas, Universidad Carlos III de Madrid, Madrid, Spain; 3 Institute for Biocomputation and Physics of Complex Systems, Zaragoza, Spain; 4 UC3M-BS Institute of Financial Big Data (IFiBiD), Madrid, Spain; 5 National Biotechnology Centre (CSIC), c/ Darwin 3, Madrid, 28049 Spain

**Keywords:** Adaptive landscape, Genotype-phenotype map, Neutral networks, Functional promiscuity, Phenotype size, Environment

## Abstract

**Background:**

Wright’s metaphor of the fitness landscape has shaped and conditioned our view of the adaptation of populations for almost a century. Since its inception, and including criticism raised by Wright himself, the concept has been surrounded by controversy. Among others, the debate stems from the intrinsic difficulty to capture important features of the space of genotypes, such as its high dimensionality or the existence of abundant ridges, in a visually appealing two-dimensional picture. Two additional currently widespread observations come to further constrain the applicability of the original metaphor: the very skewed distribution of phenotype sizes (which may actively prevent, due to entropic effects, the achievement of fitness maxima), and functional promiscuity (i.e. the existence of secondary functions which entail partial adaptation to environments never encountered before by the population).

**Results:**

Here we revise some of the shortcomings of the fitness landscape metaphor and propose a new “scape” formed by interconnected layers, each layer containing the phenotypes viable in a given environment. Different phenotypes within a layer are accessible through mutations with selective value, while neutral mutations cause displacements of populations within a phenotype. A different environment is represented as a separated layer, where phenotypes may have new fitness values, other phenotypes may be viable, and the same genotype may yield a different phenotype, representing genotypic promiscuity. This scenario explicitly includes the many-to-many structure of the genotype-to-phenotype map. A number of empirical observations regarding the adaptation of populations in the light of adaptive multiscapes are reviewed.

**Conclusions:**

Several shortcomings of Wright’s visualization of fitness landscapes can be overcome through adaptive multiscapes. Relevant aspects of population adaptation, such as neutral drift, functional promiscuity or environment-dependent fitness, as well as entropic trapping and the concomitant impossibility to reach fitness peaks are visualized at once. Adaptive multiscapes should aid in the qualitative understanding of the multiple pathways involved in evolutionary dynamics.

**Reviewers:**

This article was reviewed by Eugene Koonin and Ricard Solé.

## Background

Ithaca, New York, summer of 1932. Over five-hundred scientists from 32 countries, travelling at their own expense, met at the Sixth International Congress of Genetics. The genetist Edward Murray East organized a session on evolution where Nicolai I. Vavilov, Ronald A. Fisher, John B. S. Haldane, and Sewall G. Wright were the invited speakers. They were asked to give a non-mathematical presentation of their results, a request that forced Wright to come up with a qualitative description of his shifting balance theory [1]. The result was an enduring metaphor that has shaped evolutionary thinking [2, 3], and even some of the problems addressed by evolutionary theory, in the last eighty years: the adaptive (fitness) landscape.

Wright’s landscape represented such a severe abstraction of the whole theory behind that it necessarily had to leave aside some features, a fact that made even Wright unconfortable. He recognized the inadequacy of a two-dimensional representation of a space of very high dimensionality, and was worried about the possibly many local maxima [4]. Another difficult aspect of a static picture was its inability to capture environmental changes, in his own view. Among others, static landscapes cannot depict adaptation as a non-equilibrium response to changes in selection [5]. However, the idea of a physical landscape where populations would move and adapt following “natural” directions of improvement was strong and extremely inspiring. Variables along the axes of the plane interchangeably stood for the frequency of alleles in a population or for genotypes [6], and were soon extended to represent phenotypes, with fitness in the vertical axis [7].

By now, the image of a relatively smooth landscape, where populations adapt by going up-hill once they fix an advantageous mutation, are trapped in mountain peaks and remain isolated from other possibly higher fitness maxima by deep valleys, often appears as *the* way in which adaptation proceeds. Advances in our knowledge of the molecular structure of populations have added worries to Wright’s original concerns, resulting in a steady increase of critical views of how an up-to-date, useful and more realistic adaptive landscape should be depicted.

Important topographical elements missing in most adaptive landscapes are ridges, though empirical evidence reveals that they are remarkably common. Ridges in a two-dimensional landscape translate into neutral or quasi-neutral networks of genotypes in high dimensional systems. For *common phenotypes*, these networks might span the whole genome space. The existence of genotype networks that should make genotype spaces navigable was already hypothesized by J. Maynard Smith long ago [8], subsequently revealing as ubiquitous in models [9–12] and empirical studies [13–15] of how genotypes map onto phenotypes. An attempt to include this evidence in a landscape-like picture was made (and before the empirical evidence was so overwhelming as it is now) by S. Gavrilets proposing holey adaptive landscapes [16]. Holey landscapes, however, are still misleading regarding the actual distance between genotypes, which appear close to each other in that low-dimensional representation. Actually, surfaces in holey landscapes should be better understood as areas of relatively dense networks of phenotypes with similar fitness [17].

In addition to the controversial aspects raised up to date, there are two other features of the genotype-phenotype (GP) map of relevance in explaining the adaptive dynamics of populations which have as yet not been considered in visual metaphors of the evolutionary process. The first one is the very uneven size of phenotypes, measured as the number of genotypes that yield the latter: a few phenotypes are very common and many phenotypes are rare; the mutual accessibility of two phenotypes is moreover asymmetric. The second one reflects that the GP map actually entails a many-to-many correspondence: genotypes are plastic and may yield different phenotypes (or the same phenotype might perform more than one function) when expressed in different environments. This latter case seems to be much more common than previously thought, meaning that exaptation [18] or, at the molecular level, co-option of promiscuous, secondary gene functions [19] are likely common ways of adapting to environmental changes.

A pictorial metaphor of the adaptive process not only helps to think about adaptive dynamics, but is necessary in order to communicate qualitative features of the evolutionary process beyond the specialist community –that same request raised by East to the speakers of his session in 1932. We here propose a renovated picture in the form of an *adaptive multiscape*. It contains some of the overall traits of Wright’s classical proposal and subsequent reformulations, but also incorporates the extended, quasi-equal fitness regions of holey landscapes, the skewness of phenotype size distributions, the absence of a visual distance between genotypes, and functional promiscuity. Adaptive multiscapes are defined in a precise environment that changes at large evolutionary time scales (thus allowing mutation fixation). In this sense, they aim at offering a dynamic picture of the relationship between genotype, phenotype and fitness.

## Elements of adaptive multiscapes

Before embarking on designing an updated metaphor for genotype-to-fitness landscapes, we wish to discuss certain features relevant in the adaptation of molecular populations and absent in classical landscapes. We do not contend here whether one or another element determines the evolutionary fate of a particular population or whether any of those elements can be discarded when interpreting specific examples. Our purpose is limited to including features whose relevance is appropriately supported through current evidence.

### Genotype networks

The existence of extended networks of genotypes of quasi-equal fitness appears as an unavoidable result in genotype-to-fitness spaces of high dimensionality. A simple reasoning to understand the origin of ridges, or hypersurfaces of equal fitness, just needs to consider the likelihood that the fitness of a particular genotype is not changed by a point mutation. If, on average, genotypes yielding the same phenotype accept one or more mutations without changing fitness (i.e. have one or more neutral neighbours) the corresponding phenotype typically percolates the space of genotypes [20]. Usually, the larger the sequence the higher the probability of having at least one neutral neighbor. Thus, the condition of navigability of the space of genotypes hypothesized by Maynard Smith [8] as a requirement for efficient adaptation may hold in very generic situations.

More realistic models where genotype and phenotype can be unambiguously defined have demonstrated the ease to evolve along neutral paths in the space of genotypes. An early example was provided by populations of RNA sequences where the minimum free energy secondary structure was used as a proxy for phenotype: here, neutral evolution permits an efficient exploration of the space of phenotypes [9, 21]. Accurate models for protein folding revealed that the similarity between sequences in the obtained neutral networks is close to randomness, thus implying that neutral evolution again permits to traverse the space of genotypes [10, 22]. In other models where the fraction of non-viable genotypes is large, extended genotype networks still do exist and allow navigability, as for metabolism [12] and gene regulatory networks [11]. In all cases, movement along neutral paths grants access to an ever growing number of different phenotypes one or a few mutations away. Additionally, analyses of the presence of neutral mutations in natural systems reveal a relatively high frequency of mutations with imperceptible effects in fitness [23], thus indirectly supporting the existence of genotype networks.

### Phenotype size distribution and phenotype accessibility

Among the few examples of genotype spaces fully mapped to its corresponding phenotypes, the secondary structure of RNA sequences [24–26] and the hydrophobic-polar (HP) model for protein folding [27, 28] stand out. Those studies have permitted to gather an accurate knowledge of the distribution of phenotype sizes and the contacts between phenotypes. While all RNA molecules with known function belong to very large phenotypes [26, 29], most phenotypes seem to have smaller sizes [26, 30]. But since the distribution of sizes is very skewed, the vast majority of genotypes do belong to that small fraction of huge phenotypes. As a result, while common structures are easily found through random searches in genotype space, sequences folding into rare structures often need to be designed [22, 27, 31]. Large phenotypes are more robust to mutations [32, 33] as well, a property involved in independent effects such as the survival of the flattest [34, 35]. Simple models of protein folding reveal that a skewed distribution should also describe the abundance of protein folds [36], and more abstract GP maps sharing basic constructive rules with natural molecular systems consistently present a very broad distribution of phenotype sizes [33, 37]. Available evidence thus supports the idea that the latter is to a large extent a universal property of realistic GP maps [38].

The preference for large phenotypes, the high dimensionality of the genotype space of molecular sequences, and the fact that their associated genotype networks easily traverse the space of genotypes represents an a priori guarantee of the mutual accessibility of most common phenotypes through point mutations. There are several empirical examples that show the apposition of pairs of genotype networks. For instance, two point mutations suffice to fully exchange the molecular structure and catalytic activity of two ribozymes [13]; neutral drift in proteins gives access to new phenotypes and is able to modify the fitness of incidental, secondary phenotypes [14], thus facilitating functional evolution; in influenza, immunological escape and the concomitant finding of new infective phenotypes have been shown to take place through neutral paths [15]. All in all, non-adaptive processes such as neutral search have certainly played a main role in the evolution of biological complexity [39].

The notion of nearness, and therefore accessibility, between phenotypes as a result of the existence of neutral networks in high dimensional spaces has been extensively worked out for the RNA folding model [40, 41]. In general, the mutual accessibility of two phenotypes is not symmetric. This means that it may be easy for a population to jump from phenotype A to phenotype B, while the move from B to A is difficult. This asymmetry stems from how genotypes of a given phenotype are connected to neighboring genotypes (thus other phenotypes) [42]. Consider a phenotype that can be obtained from a unique genotype. Any mutation thus leads to a new phenotype, and some of those alternatives might have large phenotypic size. Once in the new phenotype, mutations are likely to conserve it, but at the same time separate the population from the initial phenotype. In some situations, if one of the phenotypes is sufficiently rare, it might be never found through random searches, as stated, or the typical time to attain it is so large that it is never reached in practice [43].

### Functional promiscuity

The studies reported in the two previous sections focused on the analysis of the many-to-one structure of the GP map. However, there is abundant evidence that this relationship is actually many-to-many, and the ability of genotypes to yield, in a variety of manners and under different situations, more than one phenotype (one-to-many), is a crucial property in the adaptation of molecular populations. Basic features of the one-to-many GP relationship have been described for RNA sequences. Actually, the minimum-free-energy folded state of an RNA sequence is one of several-to-many different states visited by any sequence at any finite folding temperature [44, 45]. Under different environmental conditions (as temperature or pH changes, for example), the same sequence can yield a different structure –and, in principle, also a different function. The plasticity of RNA sequences regarding their folded states is remarkable. For instance, it has been shown that any pair of RNA secondary structures can be in principle realized by properly designing a unique sequence that has those two structures as compatible folds [46]. Therefore, the properties of RNA secondary structure neutral networks do not only permit the contact (separated by one mutation) between almost any two secondary structures; these networks overlap sufficiently so as to yield any two different folded structures with one genotype. Natural selection has taken advantage of the plasticity of the RNA genotype in the design of RNA switches [47] or in a case where a sequence is reused to eventually perform three different catalytic roles in vivo [48].

There are abundant observations of functional promiscuity in other molecular systems [49]. One of the most dramatic cases is that of enzymes recruited to perform a structural function as lens proteins [50]. Another example is enzyme promiscuity, a property acknowledged and described long ago [51] that refers to the ability of an enzyme to fortuitously catalize a reaction other than that for which it evolved. This is to say, there are functions different from the main one that emerge in an unselected manner. The adaptive advantages conferred by this feature are difficult to overstate [52]. Under environmental changes or the appearance of dysfunctional genes, for instance, functional promiscuity may confer certain degree of pre-adaptation for free, or may buffer the effects of misfunctional proteins. Occasionally, the secondary function might become primary through subfunctionalization, which occurs when a duplicated gene splits its main and promiscuous functions between the two copies. Subfunctionalization seems to be a leading mechanism to maintain duplicated genes [19]. Recent models of genotype to phenotype involving whole metabolic systems [53] or intermediate levels in the expression of the phenotype [54] come to support the commonness of functional promiscuity at a systemic level.

Empirical cases where functional promiscuity has been described link in a very appealing way genotype networks and accessibility to new functions. On the one hand, evolutionary improvement of the promiscuous function can occur through the fixation of mutations neutral to the primary function but advantageous to the secondary activity [52]. On the other hand, neutral drift and the concomitant exploration of the genotype network entails the serendipitous discovery of secondary activities [14, 55]. All that evidence strongly suggests that heterogeneous molecular populations are endowed with functions unseen in the current environment that show up when conditions change.

## Adaptive multiscapes

A visual metaphor that aims at capturing relevant features of molecular evolution, as described so far, should integrate information on genotype networks and their skewed distribution of sizes, on the mutual attainability of genotypes (through mutations) and phenotypes (through mutation or promiscuity, and conditioned to their internal networked structure), and on the relationship between fitness (the environment-dependent value of phenotypes) and adaptation.

**Fig. 1:**
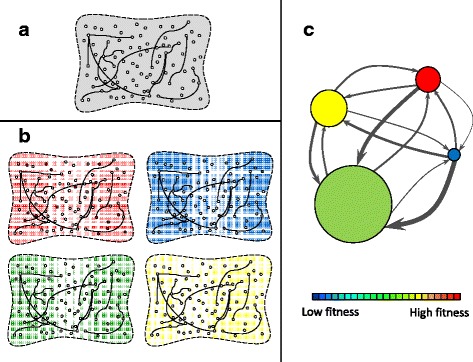
Basic elements in the construction of the adaptive multiscape metaphor. **a** Schematic representation of the space of genotypes. Two genotypes are linked if they are at a distance of one mutational move. **b** Unfolding of the genotype space into different phenotypes in a given environment. Each group contains all genotypes (*colored*) that yield the same phenotype. Links joining two genotypes in the same phenotype permit neutral evolution; links joining genotypes in different phenotypes (not explicitly shown) represent mutational moves causing changes in the phenotype. Only the network structure matters to describe population dynamics. **c** Synthetic representation of genotype networks as *circles* with area proportional to phenotype size. Thickness of *arrows* between pairs of phenotypes represents the likelihood to attain the target phenotype conditional on being on the departure phenotype. Those links are asymmetric and weighted. In a given environment, the fitness of each phenotype is color coded, from *low (blue)* to *high (red)*

Figure 1 depicts the main elements of adaptive multiscapes. The space of genotypes is first represented as an ensemble of dots (each corresponding to a genotype) mutually linked if they are at a distance of one mutational move (Fig. 1 a). (It is common to consider the “mutational move” as a point mutation, but this is not a requirement for this representation to be valid; it might be a deletion or a duplication of a genotype fragment, for instance, and the scheme remains unchanged.) The mutational move is the only notion of “distance” relevant in this representation. The space of genotypes is therefore endowed with a network structure and the two-dimensional projection becomes irrelevant as far as genotype or phenotype accessibility is concerned. Second, the full genotype space unfolds into an ensemble of phenotypes (Fig. 1 b): *given an environment* each genotype is mapped into one or a few phenotypes. Genotype networks are defined within each phenotype as a subset of genotypes and links of the whole genome space. In the figure, genotypes within a phenotype are colored, and color stands for fitness (see Fig. 1 c); only links joining genotypes in the same phenotype belong to the genotype network and permit neutral evolution. Figure 1 c synthesizes several elements of the representation: phenotype sizes, networked structure, asymmetry of phenotype accessibility, high mutual accessibility between any phenotype pair, and phenotype fitness (through color). The microscopic structure of phenotypes as heterogeneous networks of genotypes is now implicit.

**Fig. 2:**
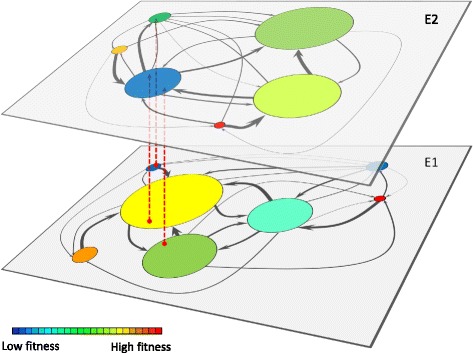
Cartoon of the mapping of a genotype space into phenotypes in two different environments. Each layer (E1, E2) represents an environment. In each of them, genotypes express different phenotypes with different fitness values. *Red dashed arrows* indicate possible cases of functional promiscuity: if the population contains a genotype in the region of the *yellow* phenotype (in E1) that overlaps the *blue* phenotype (in E2), the function required in E2 is performed, though with a decreased proficiency. For the sake of a clearer representation, not all possible transitions between phenotypes have been depicted

The previous representation depends quantitatively on the environment where the genotype-phenotype map is realized. When the environment changes, that map is also modified (and therefore the size of each particular phenotype), as are, in principle, phenotype fitness and the precise values of mutual accessibility. Figure 2 depicts the genotype-phenotype-fitness map in two different environments and serves as an example of how functional promiscuity can be visualized. The extension of this two-layer-two-environments representation of the genotype-phenotype-fitness map to an arbitrary number of different environments constitutes the visual metaphor of adaptive multiscapes.

## Population dynamics on adaptive multiscapes

In this section we discuss how different population characteristics intermingle with adaptive multiscapes features to yield a dynamical view of evolution.

### Population size

The exploration of the genotype space is limited in any natural population by its finite size. At any time, genotypes in the population cover but a tiny fraction of any phenotype. This limitation is alleviated, as discussed, by the conjunction of neutrality (costless navigation of phenotypes) and the high dimensionality of genotype space –which renders almost any alternative common phenotype accessible from the current one. This nonetheless, the size of a population and its mutation rate have a direct effect on the time spent on a phenotype, and on the likelihood to find evolutionary innovations. The relationship between phenotype size and phenotype robustness [33] also modulates the dispersion of the population in genotype space [56]. When the current phenotype is large, the average genotype in the population is more robust and the population is therefore more diverse, enjoying higher evolvability [57]. When translated to adaptive multiscapes, and beyond quantitative details, the dynamics of populations within phenotypes can be visualized as subsets of varying size and position. The larger the population the larger the region of the current phenotype (and possibly neighboring ones) represented in the population. Neutral drift becomes more relevant as the population size diminishes; therefore, the smaller the population size, the less deterministic should be our representation of “trajectories” within a phenotype. Typically, populations first access phenotypes through one or a few genotypes, so they are quite homogeneous. As the neutral network is explored the genotypic diversity of the population grows [9]; eventually, the population stabilizes around the regions of maximal neutrality provided no phenotype of higher fitness is found and fixed in the process.

### Mutations

**Fig. 3:**
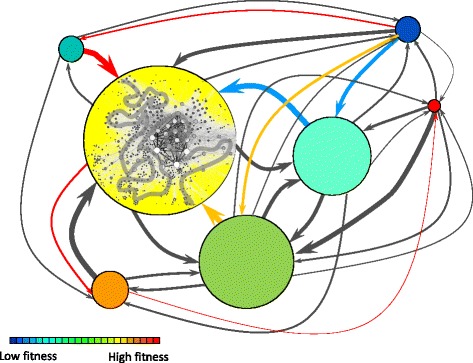
Population dynamics on adaptive multiscapes. The high degree of mutual accessibility of phenotypes is illustrated as an almost completely connected network where phenotypes are represented as *circles* with a surface proportional to the phenotype size. As depicted in the *yellow* phenotype, there is a complex, networked internal structure of genotypes, and molecular populations move on that network, though they occupy a tiny fraction of all possible genotypes. Therefore, there is a waiting time (stasis) before a fitter phenotype is found and fixed (punctuation). The *grey curve* illustrates the movement of a population within one phenotype. Different possible adaptive trajectories among phenotypes are depicted through colored links. Note that though the *red* phenotype can in principle be attained through fixation of an appropriate sequence of mutations, the time spent in the *yellow* phenotype might be, in practice, much longer than that required to find the *red* one

The effect of neutral mutations has been implicitly discussed in the previous paragraphs. Neutrality, whose absence was pinpointed early in the history of fitness landscapes, promotes navigability and the coexistence of variants within the population, and is easily visualized in our scenario. However, populations might spend a long time in the current phenotype before an adaptive move occurs [58]. Though for simplicity we visualize phenotypes as single entities, it must be kept in mind that they have a complex internal structure that affects population dynamics. Also, although any common phenotype is typically attainable in one mutational move from any other common phenotype, the precise genotypes located at the frontier of the two phenotypes might be hard to find. The appearance and fixation of mutations with an effect in fitness also have a non-trivial representation in adaptive multiscapes. In Fig. 3 we depict possible adaptive trajectories in a fixed environment. Suppose that a population begins its adaptation to that environment in the blue phenotype, which is not particularly large or fit. Sooner rather than later an advantageous mutation will appear and rise to fixation. This fact corresponds to an up-hill movement in classical Wright’s-like fitness landscapes, where beneficial changes are easily found and accumulate steadily and gradually. In adaptive multiscapes, however, the expected dynamics are different. First, there is a variable time spent on the current phenotype where mutations accumulate but no change in phenotype takes place. Second, there is a large number of fitter phenotypes accessible from the current one, and it is known that the likelihood to jump to any of them depends on two quantities at least: their fitness difference [58] and the size of the new phenotype [43]. The former feature is qualitatively captured through the change in color given by the fitness scale and the latter through the thickness of links. Third, and at odds with the dynamics implicit in Wright’s landscapes, the phenotype of highest fitness is not necessarily always found and fixed in the population, the likelihood of that event being dependent on its size [43].

Let us imagine the adaptive pathway followed by the population in Fig. 3. Advantageous mutations to six different phenotypes might occur: given the size of the yellow phenotype this transition seems likely, while the size of the red phenotype suggests it will be encountered with lower probability. Since the yellow phenotype has a fitness higher than the green one, there is no need for the population to go through that intermediate step. The previous considerations notwithstanding, any of the trajectories represented might be observed in a single realization of the process. And before any new phenotype is found, there will be a time of stasis corresponding to a random search in the current genotype network.

### Examples

After the previous discussion, which has presented in a generic fashion how the dynamic process of adaptation of molecular populations would be visualized in adaptive multiscapes, we turn to specific examples. First, we construct a synthetic example where all quantities can be exactly and unambiguously defined. We continue by rephrasing well-known empirical observations in the language of adaptive multiscapes.

#### A synthetic quantitative example

**Fig. 4:**
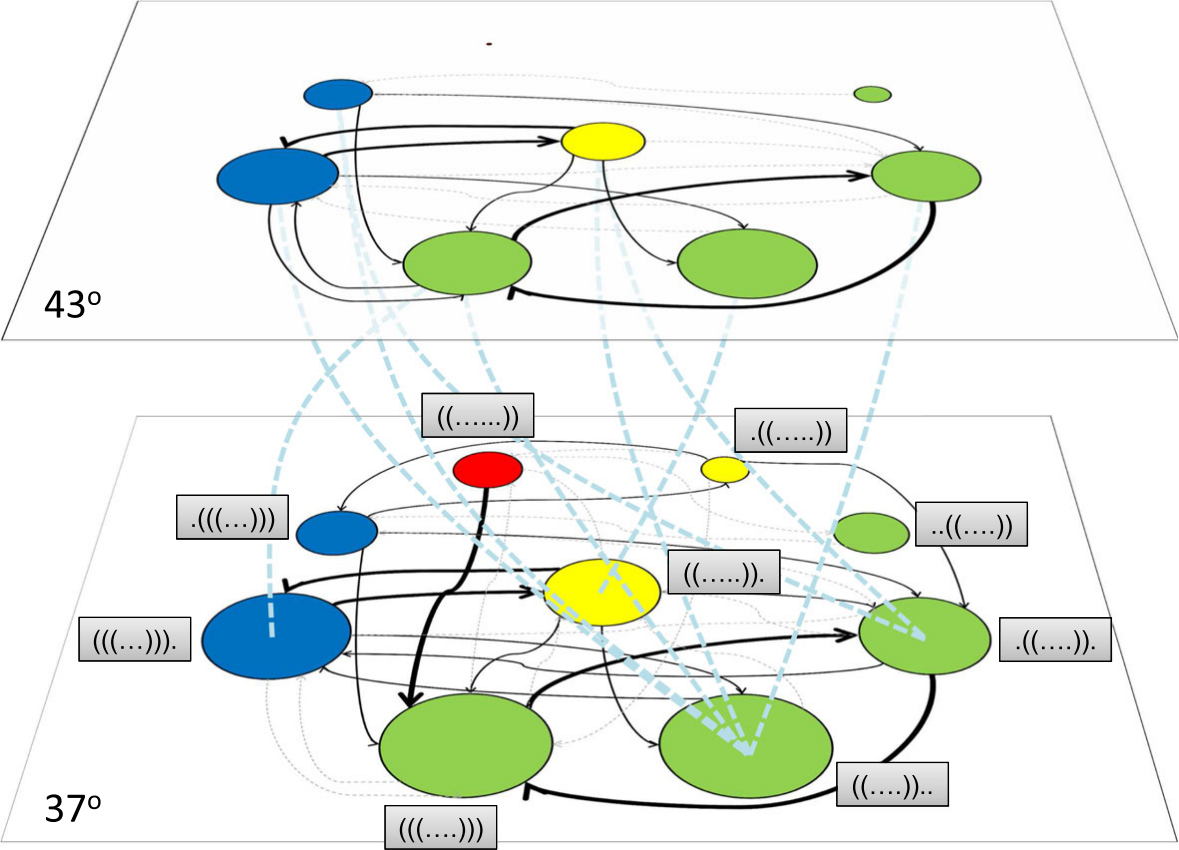
Multiscape of RNA sequences of length 10 folded at two different temperatures. There are 9 non-empty phenotypes at 37 °C and 8 at 43 °C, one of them having size 1. Fitness has been chosen to be proportional to the number of unpaired nucleotides in the hairpin loop, colors indicating fitness follow the coding scale introduced in previous figures. *Thick solid lines* between phenotypes in an environment represent a probability of changing phenotype under a point mutation above 5%; *thin solid lines* stand for probabilities between 1.5 and 5%; *dashed grey lines* are for probabilities between 1 and 1.5%; lower probabilities exist but are not shown for clarity. The largest promiscuity occurs between the same phenotype in the two environments (see Table 1); *thick dashed light-blue lines* indicate likely promiscuous transitions. Phenotypes are labeled in the 37 °C environment; they occupy the same position at 43 °C

Let us illustrate the qualitative picture presented with a complete, quantitative example of a multiscape. Consider all RNA sequences of length 10 as our space of genotypes, and the minimum energy secondary structure at a given folding temperature as the phenotype. Suppose that two different temperatures stand for two different environments, such that we can obtain a complete GP map at temperatures, e.g., 37 °C and 43 °C: the results are summarized in Table 1 and in Fig. 4. Table 1 shows the non-empty phenotypes and their size at each folding temperature, as well as the fraction of neutral mutations for each phenotype at either temperature and the probability that the phenotype is not changed under an environmental change. If we take as the original environment that at 37 °C, $p_{\text {stay}}^{37 \to 43}$ is the ratio between the sequences folding into a given phenotype at 43 °C conditional on their folding into that same phenotype at 37 °C (and similarly if the higher temperature represents the original environment). There is a large fraction of sequences that map to the open structure (i.e. they have a positive folding energy in any secondary structure). Figure 4 qualitatively summarizes the relationship between the two environments studied.

**Table 1:** Some quantitative properties of the map from RNA sequences to secondary structures at two different temperatures

Phenotype	Size at 37 °C	Size at 43 °C	$p_{\text {stay}}^{37}$	$p_{\text {stay}}^{43}$	$p_{\text {stay}}^{37 \to 43}$	$p_{\text {stay}}^{43 \to 37}$
(((...))).	6935	4307	0.3961	0.3872	0.6209	0.9998
(((....)))	7791	5149	0.3864	0.3658	0.6585	0.9963
((....))..	7766	5879	0.5142	0.4915	0.7550	0.9973
((.....)).	4802	2692	0.4431	0.4137	0.5539	0.9881
((......))	1438	1	0.4092	0	0.0007	1
.(((...)))	2287	1542	0.3843	0.3661	0.6707	0.9948
.((....)).	5718	3624	0.4470	0.3996	0.6338	1
.((.....))	944	0	0.3684	–	0	–
..((....))	1729	360	0.3861	0.2928	0.2076	0.9972

The non-empty phenotypes are listed in the first column, while the second and the third columns yield the size of the phenotype

in two different environments (at two different folding temperatures, 37 °C and 43 °C); $p_{\text {stay}}^{37}$ and $p_{\text {stay}}^{43}$ are the probabilities that

a point mutation does not change the phenotype at 37 °C or 43 °C, respectively; $p_{\text {stay}}^{37 \to 43}$ is the probability that a given sequence

folds in the same phenotype when the temperature changes from 37 °C to 43 °C, and similarly for $p_{\text {stay}}^{43 \to 37}$

As a possible definition for fitness, we have chosen it to be proportional to the number of unpaired nucleotides in the hairpin loop of the secondary structure. This definition yields four levels of fitness, as revealed by the color code. Let us imagine a population of sequences at 37 °C. The fittest phenotype is sufficiently large such that our population will be mostly found at or near that phenotype. However, due to the high likelihood of mutating from ((......)) to (((....))), we expect this second phenotype to be also populated at equilibrium. A fraction of sequences could be also found populating phenotype ((.....))., the absolute numbers depending on the population size, on the relative transition rates and on the relationship between fitness values. An increase in temperature from 37 °C to 43 °C implies a complete destabilization of the fittest phenotype. Though there is one particular sequence folding into ((......)), it cannot be reached from any other populated phenotype and any mutation leads to the open structure. In practice, the fittest phenotype will not be seen at high temperatures. A population initially in equilibrium at 37 °C has now to find its way to the new equilibrium. There are at least three possible pathways that can be followed. If there were enough sequences in phenotype ((.....))., and given the high likelihood of remaining in that phenotype under the environmental change (Table 1), adaptation could occur immediately. However, if the mutation rate or the population size were too small, (((....))) might contain all the sequences. Adaptation to the fittest (achievable) phenotype could require traversing the phenotype of lower fitness (((...))). or drifting neutrally to phenotype.((....)). at 37 °C to reach ((.....)). through promiscuous adaptation.

#### Viral populations

**Fig. 5:**
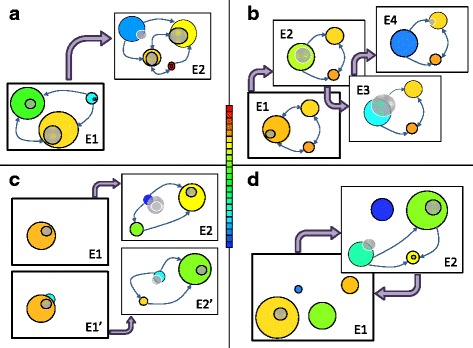
Schematic representation of examples of adaptation in the framework of adaptive multiscapes. Initial environments are labeled E1 and E1’, and different subsequent environments correspond to labels E2, E2’, E3, and E4. We use stilized representations of populations at equilibrium in an environment (*grey circles* with *black* boundary) or during adaptive transients (*grey circles* with *white* boundary). Only meaningful and likely links between phenotypes in the depicted situation are shown (note the direction of *arrows*). Large violet arrows stand for environmental transitions. **a** Distribution of viral populations. Even at equilibrium, these populations maintain high levels of genotypic and phenotypic diversity in their current environments (E1). E2 represents an environment where the population is poorly adapted initially (in the *blue* phenotype) so it needs to search the genotype space to find and fix advantageous mutations. After the initial bottleneck, it spreads over different phenotypes again. **b** Drift and switch between neutral networks. Punctuation in the phenotype is enhanced by the loss of fitness of the current phenotype, which here occurs concomitantly with the exploration (search) of the neutral genotype network. A short sequence of intermediate environments is shown (E2, E3, E4). **c** Effects of gene duplication. The fate of a duplicated gene depends on the existence of a secondary function prior to duplication. In its absence (neofunctionalization, E1, E2) the gene can acquire random mutations and explore the surrounding genotype space. If a secondary function was present (subfunctionalization, E1’, E2’), there is a viable phenotype subjected to a selection pressure that might be rapidly optimized in E2’. In both cases, evolutionary optimization begins once a function is fulfilled. **d** Waddington’s canalization of an acquired character. In the language of adaptive multiscapes, a subset of genotypes in the population expresses different phenotypes in E1 or E2. Under selection (both natural and artificial) in E2, the population modifies its genomic composition, such that if E1 is restored the population will no longer express the original phenotype (there is no overlap between the equilibrium population in E2 and the orange phenotype in E1)

Viruses, especially those with an RNA genome, maintain high population numbers and high diversity, both in genotype and phenotype. They are notorious for their fast adaptation to different environmental conditions, and especially for their ability to escape host resistance to infection or to evade sophisticated antiviral strategies. In adaptive multiscapes, viral populations appear distributed over different phenotypes and a range of fitness values [59]. In those populations, low-fitness variants might be abundant, as they are steadily generated from high-fitness variants. If the mutation rate is high enough, the fittest variant is not the most abundant one [60]. Under an environmental change, such as infecting a new host [61] or facing an antiviral therapy not experienced before [62], viruses may adapt rapidly (through advantageous mutations) or show non-adaptive viability due to functional promiscuity. These two strategies, which have important implications in the treatment of viral infections [63] find a straight representation in the visual language of adaptive multiscapes (Fig. 5 a). In either case, however, a minimum amount of viability is needed for the population to replicate and generate advantageous mutations: zero fitness implies extinction [61].

#### Stasis, genotype network search and punctuations

When a molecular population first encounters a fitter phenotype, selection for the new mutant occurs rapidly, such that genetic diversity decreases. Exploration of the genotype space follows, and the molecular diversity of the population grows as it diffuses through the genotype network. This behavior has been documented in influenza A virus [15]. Its seasonal dynamics conforms to a search and switch pattern equivalent to that described in computational populations of RNA molecules evolving towards a goal secondary structure [40]. The representation of these dynamics in the framework of adaptive multiscapes takes into account the stasis of the population during the infection season, which simultaneously expands in the genotype network of the current phenotype. As the host acquires immunity along the season, the number of susceptible individuals shrinks and the fitness of that phenotype diminishes, enhancing in consequence the possibility to jump to a new phenotype (a new antigenic cluster, Fig. 5 b).

#### Evolution of gene duplication

Two of the mechanisms proposed to favor the persistence of duplicated genes are neofunctionalization and subfunctionalization [64]. In the former case the duplicated gene has no apparent function and thus may freely accumulate mutations. The exploration of the genotype space is thus enhanced until that gene is recruited for (or finds) a new function. Subsequently, optimization under the new selective pressure might occur. In the case of subfunctionalization, the initial gene was fulfilling two functions (analogous to presenting two different phenotypes and being subject to two selection pressures). Under duplication, the two functions can be independently optimized under their respective selection pressures (analogous to two different environments). Figure 5 c illustrates the two situations.

#### Waddington’s canalization

In a series of remarkable experiments, Conrad H. Waddington [65, 66] showed how a postulated phenomenon known as genetic assimilation actually took place. Very briefly, genetic assimilation means that, under a sufficiently strong environmental change, a character that an individual only expresses in the new environment (an “acquired character”) can become “assimilated” at the genetic level: When conditions revert to the original environment, the initial phenotype is no longer expressed and the new phenotype remains. In adaptive multiscapes, this observation can be rephrased as a case of promiscuous molecular function plus neutral diffusion or adaptive improvement in the secondary phenotype (Fig. 5 d). The initial assumption, following Waddington’s observations, is that some genotypes in the initial population express one phenotype in an environment and a different one in an alternative environment. Subsequent populations in the latter change their genomic composition either through recombination [65] or through the appearance *de novo* of major mutations [66]. A different possibility, not discussed by Waddington, is that neutral mutations accumulate (the population diffuses in the genotype network corresponding to the secondary phenotype). As a consequence of either process, the population moves in genotype space and, when the conditions revert to environment 1, the original phenotype is no longer expressed.

## Discussion

As any simplified and synthetic representation of a complex process, adaptive multiscapes have inherent limitations. They are suited to capture the dynamics of molecular populations, but are not intended to describe populations of complex organisms, where developmental and regulatory processes interact with the environment to define the phenotype. Also, situations where frequency-dependent selection might be important are excluded as well, since these imply a feedback between population composition and phenotype value that cannot be a priori captured in our scenario. Finally, adaptive multiscapes are proposed as an alternative to Wrightian landscapes in adaptive situations where the high dimensionality of genotype spaces is important. For cases where only few dimensions are involved, classical landscapes might offer an accurate visualization of the molecular dynamics [67].

Though we have kept the description of adaptive multiscapes and populations dynamics mostly at a qualitative level on purpose, it is important to emphasize that the features included do have a quantitative counterpart –as illustrated by our example with short RNA sequences. Several studies have been carried out to quantify the distributions of phenotype sizes or the effect that genotype network topology has on population dynamics. Indeed, the overall topological properties of genotype networks are a subject of current interest [32]. They play a role, among others, in the attainability of evolutionary innovations [38], in the time required to reach mutation-selection equilibrium [56] and in the ticking rate of the molecular clock [58]. An exhaustive characterization of the architecture of genotype networks is a work in progress, with advances severely hampered by the astronomically large size of natural phenotypes [26, 29]. Other mechanisms could affect specific quantities in adaptive multiscapes, the look-ahead effect being a prominent example: It has been put forward [68] that errors in transcription and translation that affect the phenotype, but do not modify the genotype, might constitute an important mechanism to promote the fixation of mutations with neutral or slightly deleterious effect in fitness that are required for subsequent mutations (beneficial in the appropriate genomic context) to fix. In adaptive landscapes this effect would modify the likelihood to produce an alternative phenotype from a given one, thus promoting accessibility of phenotypes near that promiscuous one and eventual adaptation through a combination of adaptive mutations plus promiscuity (much in the sense of Waddington’s canalization or related scenarios that emphasize the adaptive role of phenotypic plasticity [69, 70]). Adaptive multiscapes have embedded that possible adaptive pathway in a qualitative manner.

Our representation has tried to emphasize how large differences in phenotype size imply that small phenotypes will be rarely visited [43]. We have been talking about “common phenotypes” to refer to those actually visited by molecular populations. Rare phenotypes are small, but the magnitude of their smallness has remained vague so far. Actually, it is a known fact that the vast majority of phenotypes are too small to be found through random searches in genotype space, and this is so independently of their fitness [26, 43]. Let us be more explicit by means of an example. In [26], the sizes of all neutral networks for RNA secondary structures of non-coding RNAs in the function RNA database [71] were measured. It was shown that all natural functional RNAs belong to phenotypes whose sizes lie in the far right tail of the probability distribution of phenotype sizes. These are common phenotypes. For example, natural, functional RNAs of length 126 nucleotides have secondary structure neutral networks of size at least 10^47^. This is about 10 orders of magnitude larger than the most abundant phenotype size, and over 20 orders of magnitude larger than those of small phenotypes. Suppose now that a population were so large as to be able of exploring the neutral network completely. In that (not just implausible but utterly impossible) case, the population would have had access to at most 3×126×10^47^≃10^49^ genotypes belonging to other neutral networks (many less actually, since many genotypes belong to the current genotype network). Since the total number of possible genotypes of length 126 is 4^126^∼10^75^, the probability that a genotype belonging to a typical (in size) phenotype is found through this procedure is of order 10^−16^. For small phenotypes that probability is as small as 10^−26^. How can we portray the minuteness of that number? All grains of sand on Earth (beaches and deserts) number about 10^19^. Finding a specific small phenotype is thus as likely as locating a precise grain of sand in ten million Earths. The situation gets worse as sequence length increases, since the difference in size between large and small phenotypes grows exponentially fast. Large phenotypes should therefore be considered as metastable solutions of the adaptive process, and the best that can be done with what is available. The adaptive process is completely blind to most phenotypes due to their rarity.

Molecular evolution is not easily reverted. The precise evolutionary trajectories followed by molecular populations are strongly contingent on the order of appearance of mutations [72–74]. This fact has an implicit counterpart in adaptive multiscapes, where the size of phenotypes qualitatively speaks for the time elapsed before a fitter phenotype is identified, and where the accessibility of the plurality of neighboring phenotypes is cast in terms of transition probabilities (the strength of links). Actually, in this metaphor the potential diversity of neutral pathways, whose similarity is strongly dependent on the topology of genotype networks [58], is not made explicit. Other studies have addressed the mean path divergence [75] (a measure of the (over-) dispersion of evolutionary trajectories when they share starting and ending points) and concluded that the smoother the landscape, the more divergent the trajectories are. This is in agreement with the relationship drawn between the heterogeneity of genotype networks and overdispersion [58] –where the endpoint is the final equilibrium state in a statistical sense. In the language of adaptive multiscapes, details on neutral evolution are not made explicit, since phenotypes are mesoscopic states of the population characterized by a (history dependent) waiting time before a new phenotype is found. They however capture the (correct) expectation that transitions would be more deterministic for lower mutation rates [76], since populations are less heterogeneous and the fixation of adaptive steps becomes more hierarchical and less contingent. However, if we aim at a full quantitative characterization the metaphor presented in this work should be complemented with microscopic descriptions of the evolutionary process [58, 75, 76].

In the language of adaptive multiscapes, the potential plurality of adaptive pathways at the level of phenotypes (the diversity of neutral pathways is implicit) is easy to visualize. If quantitative characterizations of the landscape are available, the likelihood that one or another adaptive pathway is followed can be established. Also, this metaphor reveals how the restoration of an environment does not imply that the mutational path will be undone. Hysteretic processes might be thus common in molecular evolution, and should be kept in mind whenever we wish to infer the effect of environmental changes in the genomic composition of populations [77].

## Conclusions

We have devised an up-to-date metaphor that is constructed through the integration of important features of molecular populations unknown at the time when Sewall Wright proposed his adaptive landscapes. In adaptive multiscapes, features such as neutral drift, contingency, (asymmetric) phenotypic accessibility, entropic trapping or the many-to-many nature of the genotype-phenotype relationship are visually captured in a qualitative manner, and adaptation can be portrayed as a non-equilibrium process under environmental changes. We have rephrased specific examples in the visual scenario of adaptive multiscapes with the goal of helping in the interpretation of further cases, in particular by keeping in mind alternative evolutionary pathways.

## Reviewers comments

### Reviewer’s report 1: Eugene Koonin, NCBI, NLM, NIH, USA

#### Reviewer summary

This is a very interesting, timely, perceptive and easy to read presentation of new ideas on presentation of fitness landscapes, the key metaphor and heuristic tool of evolutionary biology. Although, much like Wright’s original effort, the paper, to a large extent focuses on representation, the ideas discussed in the paper have the potential to stimulate research in the field. Overall, I expect this to be quite a useful, widely read (and, hopefully, cited) publication.

Author’s response: *We very much appreciate the overall comments of Prof. Koonin and share his hope that this paper will reveal as a useful piece.*

#### Reviewer recommendations to authors

To the best of my understanding, the authors accurately even if largely informally present the problems in the current landscape representation and possible solutions. I will make three small points. First, I find it quite interesting and to the point that the authors include discussion of Waddington’s work on canalization and assimilation. However, as far as I know, Waddington primarily attributed assimilation to recombination that brings together pre-existing mutations, and this indeed seems to be the primary explanation.

Author’s response: *Indeed, in his 1953 paper Waddington attributed the changes in phenotype to recombination. His hypothesis was that the multigenic nature of the assimilated character and the reduced number of generations required to observe assimilation spoke against new mutations as the responsible mechanism. In his 1956 paper, however, he suggests that assimilation occurred not “by the selection of many minor genes (...), but occurred by the fixation of a single major gene mutation that presumably arose de novo by chance”. These two hypotheses are now included in the main text. It is not straight forward to include recombination as an adaptive mechanism in adaptive multiscapes as they stand: though in principle recombination is just another mechanism to travel trough genotype space, it introduces effects such as density-dependent selection that affect the quantitative properties of the landscape, as mentioned in the Discussion.*

Second, I am wondering how is the previously observed clustering evolutionary trajectories reflected in the multilayer landscapes considered here; see: Lobkovsky AE, Wolf YI, Koonin EV. Predictability of evolutionary trajectories in fitness landscapes. PLoS Comput Biol. 2011 Dec;7(12):e1002302 and Lobkovsky AE, Wolf YI, Koonin EV. Quantifying the similarity of monotonic trajectories in rough and smooth fitness landscapes. Mol Biosyst. 2013 Jul;9(7):1627-31

Author’s response: *We have added a paragraph in the Discussion to establish how this interesting observation can be (partly) cast in the language of adaptive multiscapes, and which other elements would be needed in order to make the micro- to mesoscopic description of the adaptive process quantitatively complete. An important point here is to clarify the distinction between within phenotype dynamics (truly microscopic dynamics which depends on the topology of genotype networks and is mostly neutral) and between phenotypes (adaptive steps described in our metaphor at a mesoscopic level). Also, when the differences in fitness are not large, population size comes into play and quasi-neutral evolution becomes relevant, blurring the distinction between the two levels. That is, for smooth fitness landscapes we expect the population to be more spread in genotype space and contingency (quasi-neutral drift) to be visible in a plurality of possible mutational pathways. This expectation benefits from our knowledge on the intra-phenotype dynamics that we have formally studied in previous works (Manrubia S, Cuesta J. Evolution on neutral networks accelerates the ticking rate of the molecular clock. J Roy Soc Interface 12:20141010 (2015)).*

Finally, I would be interested to read what do the authors have to say about the look ahead effect: Whitehead DJ, Wilke CO, Vernazobres D, Bornberg-Bauer E. The look-ahead effect of phenotypic mutations. Biol Direct. 2008 May 14;3:18

Author’s response: *As we understand it, the look-ahead effect and other situations where phenotypic plasticity-like mechanisms play a role in adaptation are qualitatively included in adaptive multiscapes. These mechanisms are now discussed in the main text, where they appear together with new relevant references. When adaptive multiscapes are described in a quantitative fashion, phenotypic plasticity affects the probability to express an alternative phenotype. A difference between the look-ahead effect and phenotypic plasticity is that, in the former, the new phenotype appears as a result of post-translational errors and is expressed (in principle) in the same environment, while phenotypic plasticity is often understood as the expression of a different phenotype as a response to an environmental change, and is therefore more closely related to promiscuity as here defined.*

#### Minor issues

This paper has been submitted as a Research article. However, as far as I can see, there is formally no new analysis reported. I wonder whether it would be more appropriate to reclassify this as a Review or Opinion, in which case some restructuring would required.

Author’s response: *We agree with Prof. Koonin that this paper would fit better as an Opinion piece.*

### Reviewer’s report 2: Ricard Solé, ICREA, Universitat Pompeu Fabra, Spain

#### Reviewer summary

This is a very interesting paper that should stimulate a broad community of researchers. The paper makes a series of relevant points concerning the deficiencies of the standard use of landscapes and suggest a general and robust approach based on the multiscape picture. I think this is a path that should be taken in the future and the paper makes a very good job in presenting the whole framework.

Author’s response: *We very much acknowledge the overall comments of Prof. Solé. Hopefully, this path will be followed by others in the near future.*

#### Reviewer recommendations to authors

The landscape picture of evolutionary dynamics is a central component of most evolutionary problems. Because of the underlying complexity of the genotype-phenotype (GP) mapping, and due to several complexities derived from mutation, population size, multiple scales or ecological context, a proper choice of the landscape metaphor is a crucial problem. Too often, we tend to ignore most of these factors in favour of a cleaner, but necessarily incomplete picture. In this respect, I think the paper by Catalán et al. will be a very useful one for a broad range of researchers, may be far beyond the examples they present. As the authors discuss in the manuscript (placing their ideas in a proper historical context) most of the literature has been using the multi peaked surface picture of evolution on fitness landscapes, despite the early warnings by Wright himself concerning the multidimensional nature of real GP topology. Several important contributions have been made over the last two decades that have deeply modified our view of GP mappings and the proper landscape pictures to be used. In this paper an additional -and relevant- suggestion is to describe the evolution on a fitness landscape in a multilayer perspective where different environments are introduced by means of different potential layers. By using this multiscape, the fact that genotypes express different phenotypes (and associated fitness scores) is naturally included within a unified framework. I think that considerable insight could be obtained in many relevant case studies by using this type of visualisation. Moreover, several important phenomena of qualitative nature, such as the presence of punctuated changes, are also easily introduced. Perhaps the paper would benefit from some more explicit examples beyond the qualitative ones that are used as illustrations. That could be done by either using some specific experimental example or by examining a given simulation/theoretical model. Examples can include in vitro experiments of viral evolution involving an environmental change (such as cell lines) and several possibilities can be used for a modelling example. Both cases would be helpful as more explicit guidelines into how to use the multiscape framework.

Author’s response: *We thank Prof. Solé for his comments, which help placing the metaphor of adaptive multiscapes in a proper conceptual context and deriving hopefully relevant consequences. Though our first intention was to present a qualitative picture of adaptive multiscapes, we agree that an explicit example aids to properly understand how the ideas here discussed get a precise quantitative counterpart. Therefore, we have worked out and added an example: the case of RNA sequences of length 10 folded at two different temperatures. Fitness has been defined ad hoc as proportional to the number of unpaired nucleotides in the hairpin loop of the secondary structure. This nonetheless, in cases where reactivity of small RNAs with other sequences are important in function, the larger the number of unpaired nucleotides the more likely a successful interaction between the two partners. Other definitions would be possible but the general picture would not be affected.*

As a final point, I think that this can be also of great help in other areas not mentioned by the authors. Within developmental biology (where the GP mapping is a major issue) similar representations could be made using gene networks and spatial patterns as the two basic components, to be complemented with environmental layers of complexity. Some timely issues within evodevo might benefit from using this extended approach to the GP problem. Similarly, the study of cancer evolution has been shifting towards related GP problems over the last decade, as we gather more and more insight into the role played by cell heterogeneity and its impact on evolvability in carcinogenesis. Here an obvious scenario where environment experiences shifts is provided by the use of diverse types of drugs which can deeply modify the fitness landscape while creating new opportunities for innovation.

Author’s response: *We very much acknowledge these suggestions for further applications of adaptive landscapes. We have been cautious in this first publication regarding the inclusion of higher complexity levels where, e.g., interactions among genes are needed to define the phenotype. While we can derive a precise quantitative representation of multiscapes at the molecular level (and eventually write down well-defined dynamical equations), we are uncertain how this qualitative-quantitative map could be realised for cells, for instance. This nonetheless, we are happy to see that the metaphor already suggests that more complex systems such as evodevo or cancer development could be cast in the form of multiscapes. Being well aware of the power of metaphors in science, we can just hope that adaptive multiscapes correctly guide our intuitions to fruitful evolutionary scenarios.*

#### Minor issues

I am not sure the paper is a standard Research piece. I leave this to an editorial decision.

Author’s response: *We agree with the appreciation of Prof. Solé. As suggested by the Editorial Board, this paper will be published as an Opinion piece.*
